# From Conspiracy to Hesitancy: The Longitudinal Impact of COVID-19 Vaccine Conspiracy Theories on Perceived Vaccine Effectiveness

**DOI:** 10.3390/vaccines11071150

**Published:** 2023-06-25

**Authors:** Camila Salazar-Fernández, María José Baeza-Rivera, Diego Manríquez-Robles, Natalia Salinas-Oñate, Malik Sallam

**Affiliations:** 1 Departamento de Análisis de Datos, Universidad Autónoma de Chile, Temuco 4813302, Chile; camila.salazar@uautonoma.cl; 2 Laboratorio de Interacciones, Cultura y Salud, Departamento de Psicología, Facultad de Ciencias de la Salud, Universidad Católica de Temuco, Temuco 4810101, Chile; dmanriquez2018@alu.uct.cl; 3 Department of Psychology, Faculty of Education, Social Sciences and Humanities, Universidad de La Frontera, Temuco 4811322, Chile; natalia.salinas@ufrontera.cl; 4 Department of Pathology, Microbiology and Forensic Medicine, School of Medicine, The University of Jordan, Amman 11942, Jordan; 5 Department of Clinical Laboratories and Forensic Medicine, Jordan University Hospital, Amman 11942, Jordan; 6 Department of Translational Medicine, Faculty of Medicine, Lund University, 22184 Malmö, Sweden

**Keywords:** vaccine confidence, vaccine conspiracy, vaccine efficacy, vaccine effectiveness

## Abstract

The embrace of coronavirus disease 2019 (COVID-19) vaccine conspiracies has been linked to vaccine hesitancy. This study aimed to investigate the relationship between COVID-19 vaccine conspiracy theories and perceived vaccine effectiveness. The study utilized a longitudinal follow-up study in which adults in Chile completed surveys in December 2020 (T1) and May 2021 (T2). The psychometric properties of the five-item instrument on conspiracy theories for the COVID-19 vaccine were evaluated using data from T1 (*n* = 578). A confirmatory one-factor structure with suitable indicators of reliability was found. The longitudinal analysis (*n* = 292) revealed that conspiracy theories about the COVID-19 vaccine in T1 were associated with lower beliefs in its effectiveness in T2. However, no significant association was found between beliefs in effectiveness in T1 and conspiracy theories in T2. The study suggests that beliefs in conspiracy theories may temporally precede beliefs in vaccine effectiveness for COVID-19. The results have implications for strategies to address vaccine conspiracy beliefs and their implementation at the public policy level.

## 1. Introduction

Conspiracy beliefs refer to a collection of ideas positing that a covert group or organization is secretly orchestrating events or manipulating information to account for certain occurrences or to achieve specific objectives [1]. Although most conspiracies are unfounded and inaccurate beliefs (a few conspiracies turned out to be true, as elaborated on by van Prooijen and Douglas in [2]), these conspiracies can provide answers to uncertainty, shaping people’s attitudes and behaviors [3,4,5,6]. These beliefs entail the tendency to endorse the explanations involving clandestine plots by powerful entities with malevolent intent rather than holding beliefs backed by credible scientific evidence that are more probable to be true [7,8,9]. Conspiracy beliefs can stem from the following motives: (1) epistemic motives to help in understanding the surrounding world; (2) existential motives to help in feeling secure in an environment; (3) psychological motives to feel gratified by defending a fragile ego; and (4) social motives to fulfill the need to have a positive image of self or a group [3,10,11,12,13,14].

Conspiracy beliefs can be classified into two categories: First, the general conspiracy beliefs (or generic conspiracy mentality), which refer to the general and vast tendency for conspiratorial ideation that is broad in scope (e.g., beliefs that governments conceal information about the death of public figures to deceive the public) [15,16,17,18]. Second, the specific conspiracy beliefs that revolve around specific themes in terms of time, place, and context (e.g., conspiracy theories related to the 7 July 2005 London bombings [19]).

Although the difference between general and specific conspiracy beliefs was not clearly delineated in the past, recent literature pointed to the significant difference between these two categories and their possible impact as a driver of attitude and behavior [4,18]. Specifically, Imhoff et al. conceived the concept of “content contamination”, where the context, target, and goal of specific conspiracy beliefs can significantly affect the measurement of such specific conspiracies [18]. Thus, the issue of content contamination should be considered in any effort to study and measure the specific conspiracy beliefs, and the distinction between the generic conspiracy mentality and specific conspiracies should be clearly delineated [17]. However, there is robust scientific evidence indicating that specific attitudes are better predictors of specific beliefs than general attitudes [18,19].

It is worth noting that the endorsement of specific medical-related conspiracy beliefs can drive detrimental health behavior among individuals holding such beliefs. Consequently, adhering to a greater number of conspiracy beliefs longitudinally predicts a higher likelihood of receiving a COVID-19 diagnosis. The standardized beta value of this effect was 0.12, which corresponds to a small effect, considering a Cohen’s *d* value of 0.30 [20,21,22,23]. In general, the embrace of specific medical-related conspiracies can drive mistrust in health professionals and health institutions, as well as discourage individuals from seeking medical help [24,25,26,27]. Examples of the negative consequences of conspiratorial ideas on health behavior were manifested during the coronavirus disease 2019 (COVID-19) pandemic and included: lower adherence to following public health preventive measures, including the use of face masks, and reluctance or resistance to get vaccinated, defined as vaccination hesitancy [28,29,30,31,32]. Thus, the harmful effect of vaccination hesitancy extends beyond the mere holding of these beliefs to involve behavior manifested in lower vaccine uptake. In Chile, it has been found that people who have conspiracy beliefs are less willing to receive vaccination in the long term. The effect size of this finding was intermediate (beta = −0.28, which is equivalent to Cohen’s effect size of 0.50). On the other hand, it has been found in this country that as beliefs in the effectiveness of vaccines increase, people’s intention to vaccinate also increases. This was a large effect, with a standardized beta value of 0.60, which is equivalent to a Cohen’s effect size of 0.90 [23,33,34,35].

Expanding on this issue, numerous studies have observed the noticeable prevalence of specific medical-related conspiracy beliefs, indicating their wide adoption in various populations [36,37,38,39]. The prevalence of health-related conspiracy ideas varies based on different study settings, time, and place; nevertheless, the pervasiveness of this phenomenon during the COVID-19 pandemic was striking [21,40]. In the context of COVID-19, conspiracies emerged immediately and involved skepticism toward the possible explanation of the causative agent, namely severe acute respiratory syndrome coronavirus 2 (SARS-CoV-2), questions regarding the swift availability of vaccines with the widespread prevalence of beliefs that these vaccines were intended to implant microchips into people for surveillance and control purposes [30,41]. For example, Freeman et al. showed that the prevalence of COVID-19-related conspiracies among the population in the U.K. could be as high as 50% [42]. Conspiracies regarding COVID-19 were also found in a quarter of the participants in a study from Croatia [43]. In Jordan, a study showed that 57% of the public surveyed believed that COVID-19 resulted from biological warfare [41]. Recent studies from the Middle East countries (Jordan and Kuwait) in the context of the recent monkeypox (Mpox) outbreak also showed that the embrace of conspiracies toward emerging virus infections was dominant among various subpopulations, including health professionals [44,45,46]. A similar trend was observed in Latin America, where it was found that lower levels of general trust predicted more beliefs in conspiracy theories, such as the intentional creation of SARS-CoV-2. The standardized beta value of this effect was 0.06, which is equivalent to a Cohen’s *d* value of 0.10 and represents a developing effect according to the Sensu Hattie interpretation [23,47]. Similarly, it has been found that beliefs about the negative consequences of the COVID-19 vaccine (e.g., the possibility of increased virus contraction or more complex effects than the virus itself) are one of the main predictors of intention to get vaccinated against COVID-19 [48]. In Chile, findings indicate a negative correlation between conspiracy beliefs and beliefs about vaccine effectiveness, as well as a positive correlation between beliefs about vaccine effectiveness and vaccination intention [49]. Specifically, cross-sectional findings indicated that people who have higher beliefs in conspiracy theories tend to have less confidence in vaccine effectiveness, reflected in a large effect size (standardized beta value was 0.61, which is equivalent to a Cohen’s *d* of 0.90). Conversely, those who have a strong belief in vaccine effectiveness showed a greater willingness to vaccinate, as evidenced by a standardized beta value of 0.74, which corresponds to a Cohen’s *d* of 0.90, signifying a large effect [23,49].

One of the specific conspiracy beliefs surrounding vaccination in general and COVID-19 vaccines, in particular, involves the vaccine’s effectiveness and safety aspects [40,50]. Vaccine confidence involves trust in vaccination and trust in health providers and health institutions [51,52]. Particularly, vaccine confidence refers to a person’s attitude of acceptance and willingness to receive a vaccine based on the belief in its safety, efficacy, and benefits. In addition, vaccine confidence can be understood as the extent to which a person believes in the science behind vaccines and is willing to vaccinate themselves or their children to prevent diseases and protect others from diseases [53]. Since vaccine confidence was previously shown to be an important psychological determinant of vaccine acceptance and uptake [54,55,56], the perception that vaccines are unsafe, dangerous, or lacking effectiveness could be linked to lower intention to get vaccinated, jeopardizing the health of the self and the community [57,58,59]. Mistrust in vaccine safety and effectiveness has been shown previously in link with conspiracy ideas [52,60], with notable examples from the Middle East for various vaccines [30,33,61,62], Latin America, and Chile [48,49]. Previous studies have shown the pervasiveness of conspiratorial ideas that involve mistrust in COVID-19 vaccination [60,63]. These ideas entail that SARS-CoV-2 vaccination aims to implant microchips into people for control purposes, as well as the belief that COVID-19 vaccination can result in infertility to reduce the global population size [30,61].

One of the major negative consequences of COVID-19-specific conspiracy beliefs is the previous evidence showing its association with vaccine hesitancy and rejection with robust effect sizes [35,49,50,64,65]. Prior to the COVID-19 pandemic, conspiracies impacted the parental acceptance of childhood vaccination, particularly the measles, mumps, and rubella (MMR) vaccine, based on the controversies surrounding the unscientific claim that such vaccines were linked with autism, besides the feeling that vaccines are ineffective or unimportant [66,67,68].

Several theoretical frameworks were utilized to decipher the issue of COVID-19 vaccine hesitancy, including the 3C, 5C, and 7C models, as well as the incorporation of the health belief model (HBM) and theory of planned behavior (TPB) [51,69,70,71]. A majority of these models suggested that vaccine confidence (including confidence in vaccine effectiveness) is a major driving factor in the intention to get vaccinated [49]. 

The scientific literature has consistently shown associations between conspiracy beliefs and negative attitudes toward vaccines. Studies have found that individuals who believe in conspiracy theories, such as the idea that vaccines are designed to harm people rather than protect them [48,49], were less likely to vaccinate and trust the safety and effectiveness of vaccines [72]. It is important to note, however, that these studies were cross-sectional and did not allow for the establishment of causal relationships. Therefore, it is unclear whether conspiracy beliefs generate negative beliefs about vaccines or whether negative beliefs about vaccines generate conspiracy beliefs. Moreover, it is possible that both beliefs are mutually reinforcing, leading to a bidirectional association. Consequently, further longitudinal research is necessary to gain a better understanding of this complex and multidirectional relationship. Relatedly, longitudinal research on conspiracy beliefs about human immunodeficiency virus (HIV) and sexual risk has shown that higher levels of HIV conspiracy beliefs were significantly associated over time with risky sexual relations. After six months, it was associated with an increased likelihood of reporting risky sexual behavior. Specifically, this study found that 54% of those who endorsed conspiracies reported having had unprotected sex [73].

Since vaccine confidence and vaccine acceptance are closely intertwined, several previous studies addressed this association with conspiracy at the center of this complex and multifaceted area of research. Nevertheless, a majority of previous studies suffered from a notable caveat considering the cross-sectional nature of these studies [32,74]. Additionally, some studies lacked proper validation of the psychometric properties of survey instruments. Thus, the current study aimed to address the aforementioned limitations by establishing the psychometric properties of vaccine conspiracy and confidence scale as well as to longitudinally assess the relationship between COVID-19 vaccine conspiracy and trust in vaccination over time.

## 2. Materials and Methods

### 2.1. Participants

A sub-sample was utilized from a project carried out by the main authors, which, using purposive non-probabilistic online sampling, reached 1497 Chilean adults. Nevertheless, complete data were successfully collected from 1.040 participants, constituting a response rate of 69.47%. From this group of 1.040 participants, a random sample of 578 participants was chosen to test our hypotheses. The primary study’s inclusion criteria were: (1) residency in Chile during the COVID-19 pandemic, (2) being at least 18 years of age, and (3) having an Internet connection to facilitate responses to the online survey. Participants’ ages ranged from 18 to 76 years old. Of these, 53.46% identified as female, and 46.53% had completed university studies or more. 

These participants were invited to partake in this study between December 2020 and January 2021 (T1), prior to the mass vaccination in Chile, which began in February 2021. In the survey, participants could provide their e-mail addresses, indicating that they wished to receive more information about the current study or wished to be contacted for a follow-up. After the mass vaccination campaign, it was decided to reach out to these participants and invite them to a follow-up to assess the stability and directionality of the measurements from the main research project. This second evaluation occurred in May 2021 (T2) amidst the ongoing mass vaccination process in Chile. Only 292 agreed to participate in this study (retaining 50.5% of the sample). Participants of T2 mostly identified with the feminine gender (70.0%) and had a mean of 34 years old (*SD* = 11.23). Details of participants’ characteristics from T1 and T2 are in (Table 1). Proportion comparison tests were conducted for each category of the sociodemographic variables between the T1 and T2 samples. No statistically significant differences were found. This indicates that, in terms of sample characteristics, the T1 and T2 samples are equivalent.

### 2.2. Survey Instrument

After providing their informed e-consent, participants were required to respond to the main study’s questionnaire as well as a sociodemographic questionnaire. It is important to note that the sociodemographic variables were assessed at the end of the main study’s survey, which accounts for more than a 25% data loss in these variables. This approach was used to prioritize responses to the study’s central variables. Given that this study is derived from that broader study, only the instruments related to the fulfillment of the objectives specified in the current research will be detailed. Specifically, participants responded to the following scales (refer to Table 2 for the items from each of the scales): 

#### 2.2.1. Conspiracy Beliefs about COVID-19 and Its Vaccine’s Scale (CBS Scale)

An instrument composed of five items was translated and adapted from Brotherton et al. [17]. These items aimed to assess conspiracy beliefs about the origins of COVID-19 (e.g., bioweapon to destabilize the world or reduce the world population size) and COVID-19 vaccine conspiracies (e.g., the vaccines have already been created, the vaccines contain a microchip to be implanted in people, the vaccines were manufactured to control people and to obtain economic profits). These conspiracy items were measured on a 5-point Likert scale, with participants indicating their degree of disagreement (1) or agreement (5) with these items. Higher scores indicated a greater belief in conspiracy theories about COVID-19 and its vaccination. This scale underwent preliminary testing using a pilot study of 149 university students. Exploratory factor analysis (EFA) and reliability studies were carried out to assess the structure of the CBS. The EFA results demonstrated that the data matrix was factorizable, Kaiser–Meyer–Olkin (KMO) = 0.768, Bartlett’s test (10) = 369.162, *p* < 0.050. The 5-item scale displayed factor loadings from 0.692 to 0.847 and accounted for 54.1% of the variance. The single-factor scale exhibited suitable reliability (ω = 0.870) (for more details about the pilot study, please see Supplementary Materials File S1). 

#### 2.2.2. Beliefs about Vaccine Effectiveness Scale

Beliefs about vaccine effectiveness were assessed using an instrument that was developed by Salazar-Fernández et al. [75] and included six items that assess beliefs about the effectiveness and usefulness of vaccines in controlling diseases. The first three items were positively worded (e.g., “By getting vaccinated, you protect others against diseases”), and the last three were negatively worded (e.g., “In general, vaccines have more negative consequences than the disease itself”). Participants had to indicate their level of disagreement (1) or agreement (5) with each statement for the first three items, while the last three items were flipped in the scoring. Higher scores indicate higher beliefs about vaccine effectiveness. A previous study [49] showed a suitable level of reliability in this one-factor scale (ω = 0.864).

### 2.3. Procedure

The participants provided their informed consent, indicating their willingness to participate. The national study was conducted using QuestionPro, with the survey distributed between December 2020 and January 2021 (T1), prior to the onset of the mass vaccination process in Chile. The Ethics Committee from the sponsoring university approved this study (N°65/20). The informed consent outlined the study’s objectives and guaranteed the participant’s anonymity, confidentiality, and contact information. Participants who wanted more information about the study or who were interested in being contacted for a follow-up provided their e-mail addresses. During April and May 2021 (T2), coinciding with the start of the mass vaccination program in Chile, the participants who had provided their e-mail addresses were contacted and invited to participate in a follow-up study (i.e., panel study). Participants were assured that their e-mail addresses would only be used to contact them and pair their responses with previously collected data. 

### 2.4. Data Analysis

Firstly, we used confirmatory factor analysis (CFA) to evaluate the model structure of the data. Since data were at an ordinal level, the model was estimated using the diagonally weighted least squares (DWLS), which is more suitable for ordinal data [76]. Model fit was assessed using the conventional fit indices: *χ*^2^ with its degrees of freedom, the comparative fit index (CFI), the Tucker–Lewis index (TLI), the standardized root mean residuals (SRMR), and the root mean square error of approximation (RMSEA) with its confidence interval at 90%. According to the conventional goodness of fit criteria, these indices were interpreted: CFI and TLI > 0.95 and SRMR and RMSEA ≤ 0.08 [77]. We also evaluated the model’s reliability using the average variance extracted (AVE) and the composite reliability (CR). An AVE greater than 0.50 and CR greater than 0.70 indicates appropriate construct consistency [78].

Secondly, descriptive analyses were performed on the data of T1 and T2, and a paired *t*-test was used to compare them. Then, we tested the model fit of each scale for T1 and T2 and tested longitudinal invariance. According to McKinnon, Curtis, and O’Connor (2022), by imposing successive restrictions on parameters, we ensure that the estimation allows meaningful comparisons between the two times [79].

Configural (restriction on the form), metric (restriction on factor loadings), and scalar (restriction on intercepts) invariance models were tested. Then, using structural equation models, we specified a cross-lagged panel model with the aim of assessing longitudinal associations between conspiracy theories and beliefs about vaccine effectiveness. Comparison between the invariance model was performed by assessing changes in CFI and RMSEA. According to Sass et al. [80] and Rutkowski and Svetina [81], if ∆CFI > 0.010 and ∆RMSEA > 0.015, we rejected model invariance.

Cross-lagged panel models estimate two parameters: (1) autoregressive paths and (2) cross paths [82]. The autoregressive paths are the path from one variable on T1 to the same variable on T2 (i.e., conspiracy beliefs on T1 to conspiracy beliefs on T2). The cross-path is the path from one variable on T1 to another variable on T2 (i.e., conspiracy beliefs on T1 to beliefs about vaccine effectiveness on T2). While autoregressive paths inform us about the stability of the measure across time, the cross paths tell us which variable can be considered an antecedent and which can be considered a consequence. This antecedent-consequence relation is also known as Granger causality (see more on Hamaker et al. [83]).

We also estimated the 95% confidence interval of each parameter (autoregressive and cross-lagged). If the confidence interval comprehends zero, the results should be considered non-statistically significant [84].

## 3. Results

### 3.1. Descriptive and Within-Person Comparisons

The participants’ descriptive data can be found in Table 2. These results demonstrate that the participants have low adherence to COVID-19 conspiracy beliefs at both T1 and T2. When performing within-person comparisons, it was found that these beliefs significantly decreased at T2. As for the results on beliefs in vaccine effectiveness, participants showed high adherence to these beliefs at both T1 and T2, in both the direct and reverse items. Specifically, in the direct items (2 and 3), beliefs in vaccine effectiveness significantly increased at T2 versus T1, while in the reverse items (i.e., those measuring beliefs in vaccine ineffectiveness), these beliefs significantly decreased at T2. It is important to note that for item 1, which refers to the belief that vaccination protects others from disease, participants did not report statistically significant differences between T1 and T2. This suggests that these beliefs remained constant before and after the COVID-19 vaccination process.

### 3.2. Confirmatory Factor Analysis for Each Scale at T1 and T2

Then, we tested the factor structures of our scales at T1 and T2. CBS reported a suitable fit to the data on both T1 and T2 (see Table 3). Specifically, T1 exhibited factors loadings ranging from 0.68 to 0.85 (see Table 2). Construct consistency for the CBS was also found to be satisfactory, with AVE = 0.614 and CR = 0.887. Table 3 also shows the fit indices for the one-factor models of the Beliefs About Vaccine Effectiveness Scale. This scale also reveals an excellent model fit at both T1 and T2.

### 3.3. Cross-Lagged Analysis

Before proceeding with the cross-lagged analysis, we tested the longitudinal invariance of the model. Three models were estimated, testing configural, metric, and scalar invariance. All changes in ∆CFI and ∆RMSEA were lower than 0.010, so longitudinal invariance was achieved. This step is crucial to estimate the cross-lagged panel model.

**Figure 1:**
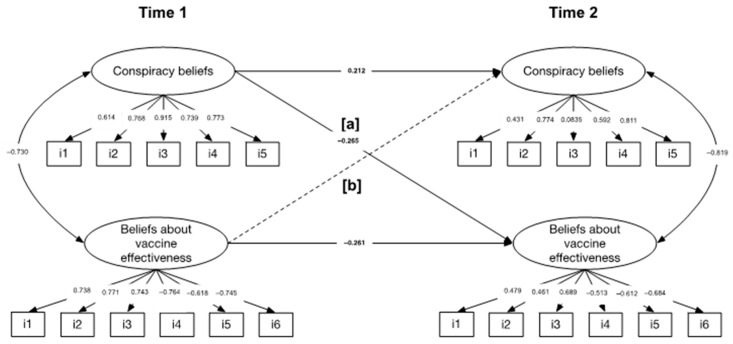
Cross-lagged panel model results. All continuous lines represent statistically significant paths (*p* < 0.050). Dashed lines are non-significant paths (*p* ≥ 0.05). “i” denotes the item number for each scale. The numbers following this letter correspond to their numbering in the scale. The items for each scale can be found in Table 2.

Cross-lagged panel model also showed a suitable model fit, *χ^2^* (214) = 220.937, *p* = 0.358, CFI = 0.997, TLI = 0.997, SRMR = 0.080, RMSEA = 0.012 (90% CI: 0.000–0.031). Regarding the autoregressive paths (path in the same variable from T1 to T2), we found that conspiracy beliefs and beliefs about vaccine effectiveness were statistically significant: β = 0.212, *p* < 0.050, (95% CI: 0.039–0.236), and β = −0.261, *p* < 0.050, (95% CI: −0.252 to −0.059), respectively. Although both autoregressive paths were stable, they showed a trend over time. Specifically, scores at T1 for conspiracy beliefs predicted higher scores for conspiracy beliefs at T2, while scores at T1 for beliefs about vaccine effectiveness predicted lower scores at T2. More importantly, we found that the path from conspiracy beliefs on T1 to beliefs about vaccine effectiveness on T2 was statistically significant: β = −0.265, *p* < 0.050, (95% CI: −0.262 to −0.057), whereas the cross-path from beliefs about vaccine effectiveness on T1 to conspiracy beliefs on T2 was not: β = 0.138, *p* = 0.065, (95% CI: −0.006 to 0.183). Covariances between conspiracy beliefs and beliefs about vaccine effectiveness were statistically significant at T1 (β = −0.730, *p* < 0.050, 95% CI: −0.575 to −0.460) and at T2 (β = −0.819, *p* < 0.050, 95% CI: −0.251 to −0.185). This means that higher scores on conspiracy beliefs are associated with lower scores on beliefs about vaccine effectiveness for T1 and T2. See Figure 1 for the graphical representation of autoregressive and cross paths.

## 4. Discussion

The current study offers psychometric evidence for a scale that assesses specific conspiracy beliefs related to COVID-19 and examines the temporal relationship between these beliefs and the perceived vaccine effectiveness using a longitudinal follow-up study. The primary finding indicated that embracing COVID-19 vaccine conspiracy theories is a robust predictor of perceived vaccine effectiveness and not vice versa.

Regarding the descriptive data, the within-person comparison revealed that conspiracy beliefs about COVID-19 were initially low and then significantly decreased between T1 and T2, a period during which mass vaccination began in Chile. On the other hand, beliefs in the effectiveness of vaccines were high at T1, and, for most of these items, these beliefs either significantly increased at T2 or remained stably high.

In the first study, we present strong psychometric evidence concerning the conspiracy beliefs about the COVID-19 scale. Specifically, this 5-item scale showed appropriate construct fit, high internal consistency, and satisfactory model reliability. These findings suggest that the scale is a reliable and valid instrument for assessing conspiracy beliefs about COVID-19. Consequently, researchers can use this scale to evaluate and gain a better understanding of the prevalence of these beliefs within different populations or their association with other health outcomes. 

Then, we addressed a limitation in the vaccine literature by adopting a longitudinal design to investigate the directionality of conspiracy beliefs on beliefs about vaccine effectiveness. This represents a novel approach, as much of the literature addressing COVID-19 vaccine hesitancy has primarily relied on cross-sectional designs, which inherently possess the limitation of not being able to infer such causal relationships [32,74]. More importantly, we found a one-way negative longitudinal association between conspiracy beliefs and beliefs about vaccine effectiveness. We did not find support for a reciprocal relationship, meaning that beliefs about vaccine effectiveness did not predict subsequent conspiracy beliefs. This result suggests that conspiracy beliefs have the potential to influence beliefs about the vaccine’s effectiveness, but not vice versa. This is consistent with previous literature that has demonstrated a correlation between specific medical-related conspiracy beliefs and perceptions regarding vaccine effectiveness, particularly in the context of COVID-19 [20,22,85]. Studies have shown that endorsing specific vaccine conspiracy beliefs is correlated with a negative attitude toward vaccination, as reflected by lower intention to get vaccinated and lower vaccine uptake [33,40,50]. This association seems reasonable considering that specific vaccine conspiracies involve the belief that vaccines are ineffective and are manufactured for the profit of Big Pharma. 

Concerning beliefs about vaccine effectiveness as a health outcome in this study, it is essential to note that several studies that have pointed out the importance of vaccine confidence, including trust in vaccine effectiveness as a crucial predictor of vaccine acceptance [86,87,88,89]. In the current study, the findings revealed an association between the embrace of conspiracy beliefs with perceived vaccine effectiveness. Thus, conspiracy beliefs are indirectly associated with vaccine acceptance, which can have a significant impact on public health by contributing to vaccine hesitancy and compromising safe population immunity [37,90,91]. 

Identifying the longitudinal negative association between conspiracy beliefs and beliefs about vaccine effectiveness serves as a starting point for developing targeted interventions to address conspiracy beliefs, which can improve the public perception of vaccine effectiveness. Furthermore, understating the causal mechanisms that drive this negative association offers valuable insights for designing public health messaging and communication strategies. These strategies can emphasize evidence-based information about vaccine effectiveness, debunk conspiracy theories, and address public concerns.

Another relevant result was the finding that both beliefs (conspiracy and vaccine effectiveness) increased over time. This could reflect that the spread of conspiracy theories increased during the pandemic, particularly with non-traditional media such as social media platforms [92,93]. In this regard, it is important to consider that the longitudinal sample had an average age of 34 years, making it the second most frequent age group in Chile for social network consumption, following emerging adults (18 to 30 years old) [94,95,96]. This may represent a bias when interpreting the information regarding the pandemic and explaining the worsening of both beliefs over time. Additionally, the fact that both beliefs have increased could be linked to the self-confirming and generalizable nature of social schemas, which are the basic units we use to interpret social information [97]. This phenomenon has been called the “perseverance effect” [98] and suggests that when information is ambiguous or contradictory (a situation that could occur when exposed to various media), the tendency is to confirm previous social schemes (e.g., beliefs about the effectiveness of vaccines), preventing their change and even exacerbating them. 

Building on this idea, the adoption of vaccine conspiracy beliefs has the potential to undermine the perceived effectiveness of vaccines in several ways: First, conspiracies have been linked to mistrust in health professionals and institutions [60,99,100]. Thus, the communication of vaccine efficacy and effectiveness data would be less effective among people who hold conspiracy beliefs. Second, conspiracy beliefs can lead to seeking information from non-credible sources, further augmenting the perceived lack of effectiveness of vaccination (echo chambers) [101]. Given the results obtained in this study and the potential consequences that erroneous information can have on people’s health, regulating the veracity of the information on social networks emerges as a significant challenge, which involves addressing ethical and legal concerns. This not only entails preventing the circulation of false information and educating the population to identify it but also promoting the dissemination of scientific evidence among the general population emphasizing the effectiveness of vaccines and how they are associated with less severe diseases and lower mortality rates. 

### Study Limitations and Future Perspectives

Despite obtaining solid results in the present study, future research needs to address several challenges. First, researchers should carefully consider sample characteristics to ensure the generalizability of the results. As such, the findings presented in this study can only be generalized to sample characteristics. Second, the COVID-19 pandemic and online data collection have posed problems that limit the inclusion of individuals without Internet access. Consequently, future studies should explore data collection alternatives that allow participation from all segments of the population. Third, it is important to note that this study was conducted before (T1) and after the start of mass vaccination (T2), so the results presented are applicable only to this period. Future studies should assess whether these beliefs remain stable over time, considering the number of vaccine doses that have emerged for protection against COVID-19. Fourth, having only two time points limits the use of more advanced longitudinal models, such as random-intercept cross-lagged panel models. Two times cross-lagged models have several drawbacks regarding the disaggregation of the variance within and between persons [83]. Future studies should consider having at least three time points. 

Based on the limitations, opportunities arise to explore new areas of research. One area should involve the investigation of other variables related to vaccination behavior. Specifically, given the identified longitudinal association between conspiracy beliefs and beliefs about vaccine effectiveness, future studies should delve deeper into the social and cultural variables that might influence the development and maintenance of these beliefs, as well as their relation to vaccination decisions and other health behaviors, such as mask usage. Furthermore, it is essential to enhance and adapt emergency communication strategies related to COVID-19 vaccination. The implementation of accurate and effective communication strategies can play a pivotal role in controlling and promoting health-related behaviors. Therefore, future studies should focus on experimental designs aimed at testing and creating clear, accessible, and persuasive messages that counteract conspiracy beliefs and foster trust in vaccine efficacy. Such efforts could significantly impact the formulation of effective public health policies. 

Finally, future research should focus on deepening the understating of the causal mechanisms behind the association between conspiracy beliefs and beliefs about vaccine effectiveness. By doing so, we can work toward fostering greater trust in vaccines and ultimately safeguarding public health during the ongoing COVID-19 pandemic and beyond. 

## 5. Conclusions

The current study provides evidence on psychometric properties for a scale that assesses specific conspiracy beliefs related to COVID-19 and demonstrates that these beliefs are longitudinally associated with beliefs about vaccine effectiveness but not vice versa. These findings highlight the importance of addressing conspiracy beliefs to improve the public perception of vaccine effectiveness and, consequently, vaccine acceptance.

## Figures and Tables

**Table 1:** Sociodemographic characteristics of the samples used in T1 and T2.

Variable	T1 Sample *n* = 578	T2 Sample *n* = 292	Proportion Comparison T1 vs. T2
Gender identification			
Feminine	309 (53.46%)	151 (51.71%)	*χ*^2^ (1) = 0.23, *p* = 0.62
Masculine	113 (19.55%)	64 (21.91%)	*χ*^2^ (1) = 0.65, *p* = 0.41
Other	3 (0.51%)	1 (0.03 %)	*χ*^2^ (1) = 1.24, *p* = 0.26
Not answered	153 (26.47%)	76 (26.02%)	*χ*^2^ (1) = 0.02, *p* = 0.88
Age (years)			
Mean (standard deviation)	36.09 (13.94)	34.03 (11.23)	-
Educational level			
Completed high school	49 (8.47%)	22 (7.53%)	*χ*^2^ (1) = 0.22, *p* = 0.63
Ongoing university studies	106 (18.33%)	61 (20.89%)	*χ*^2^ (1) = 0.80, *p* = 0.37
Completed university studies	149 (25.77%)	79 (27.05%)	*χ*^2^ (1) = 0.16, *p* = 0.68
Uncompleted and completed graduate studies	120 (20.76%)	54 (18.49%)	*χ*^2^ (1) = 0.60, *p* = 0.43
Not answered	154 (26.64%)	76 (26.02%)	*χ*^2^ (1) = 0.03, *p* = 0.84

**Table 2:** Items, factor loadings from confirmatory factor analysis, and within-person comparisons of the scales.

Conspiracy Beliefs about COVID-19	Factor Loadings from CFA (T1)	Within-Person Comparisons		
		T1 (M, SD)	T2 (M, SD)	Paired *t*-Tests
1. COVID-19 is a biological weapon created by some countries to destabilize the world	0.84	2.30 (1.28)	1.77 (1.09)	*t*(242) = 5.18, *p* < 0.05
2. COVID-19 was created to reduce the world population	0.85	2.18 (1.24)	1.71 (1.02)	*t*(242) = 4.79, *p* < 0.05
3. Vaccine against COVID-19 will have a microchip to control persons	0.68	1.57 (0.87)	1.16 (0.50)	*t*(242) = 6.38, *p* < 0.05
4. Vaccine against COVID-19 was already created, but they are retaining it to maintain people controlled	0.74	1.95 (1.09)	1.45 (0.86)	*t*(242) = 5.75, *p* < 0.05
5. Big Pharma created COVID-19 to benefit from vaccines	0.77	2.09 (1.13)	1.74 (1.04)	*t*(242) = 3.71, *p* < 0.05
**Beliefs about Vaccine Effectiveness Scale**		**Within-Person Comparisons**		
		**T1 (M, SD)**	**T2 (M, SD)**	**Paired *t*-Tests**
1. By getting vaccinated, you protect others against diseases	-	4.18 (1.08)	4.36 (1.04)	*t*(225) = −1.74, *p* = 0.08
2. If vaccines have been tested, they should be utilized	-	4.21 (0.99)	4.65 (0.62)	*t*(225) = −5.58, *p* < 0.05
3. Vaccines have been a significant mechanism for reducing the spread of infectious diseases	-	4.55 (0.82)	4.71 (0.73)	t(225) = −2.11, *p* < 0.05
*4.* *Vaccines increase the likelihood of me becoming ill*	-	1.97 (0.98)	1.54 (0.86)	*t*(225) = 4.77, *p* < 0.05
*5.* *In general, vaccines have more negative consequences than the disease itself*	-	1.73 (0.94)	1.35 (0.63)	*t*(225) = 5.21, *p* < 0.05
*6.* *I believe that it is better to gain immunity naturally than through a vaccine*	-	2.00 (1.22)	1.50 (0.85)	*t*(225) = 4.87, *p* < 0.05

Note: The items in italics are written negatively and must be recoded. M = mean, SD = standard deviation.

**Table 3:** Fit indices for the confirmatory factor analysis of conspiracy beliefs scale and beliefs about vaccine effectiveness at T1 and T2.

Model	*χ* ^2^	*df*	*p*	CFI	TLI	RMSEA (90% CI)	SRMR
Conspiracy beliefs—T1	1443.568	10	<0.001	1.000	1.000	0.000 (0.000–0.087)	0.033
Conspiracy beliefs—T2	676.359	10	<0.001	1.000	1.000	0.000 (0.000–0.075)	0.056
Beliefs about vaccine effectiveness—T1	1419.704	15	<0.001	0.999	0.999	0.024 (0.000–0.080)	0.041
Beliefs about vaccine effectiveness—T2	297.021	15	<0.001	1.000	1.000	0.000 (0.000–0.054)	0.053

Note. df = degrees of freedom.

## Data Availability

Not applicable.

## Supplementary Materials

The following supporting information can be downloaded at: https://www.mdpi.com/article/10.3390/vaccines11071150/s1, Supplementary Materials File S1: Pilot study.

## Abbreviations

AVE	Average variance extracted
CBS	Conspiracy beliefs scale
CFA	Confirmatory factor analysis
CFI	Comparative fit index
COVID-19	Coronavirus disease 2019
CR	Composite reliability
DWLS	Diagonally weighted least squares
EFA	Exploratory factor analysis
HBM	Health belief model
HIV	Human immunodeficiency virus
KMO	Kaiser–Meyer–Olkin
M	Mean
MMR	Measles, mumps, and rubella
Mpox	Monkeypox
RMSEA	Square root of the mean error of approximation
SARS-CoV-2	Severe acute respiratory syndrome coronavirus 2
SD	Standard deviation
SRMR	Square root of the standardized mean residuals
T1	Study 1—December 2020
T2	Study 2—May 2021
TBP	Theory of planned behavior
TLI	Tucker–Lewis Index
UK	United Kingdom

## References

[1] Douglas K.M., Sutton R.M. (2023). What Are Conspiracy Theories? A Definitional Approach to Their Correlates, Consequences, and Communication. Annu. Rev. Psychol..

[2] van Prooijen J.-W., Douglas K.M. (2017). Conspiracy theories as part of history: The role of societal crisis situations. Mem. Stud..

[3] Douglas K.M., Sutton R.M., Cichocka A. (2017). The Psychology of Conspiracy Theories. Curr. Dir. Psychol. Sci..

[4] van Prooijen J.W., Douglas K.M. (2018). Belief in conspiracy theories: Basic principles of an emerging research domain. Eur. J. Soc. Psychol..

[5] Clarke S. (2002). Conspiracy Theories and Conspiracy Theorizing. Philos. Soc. Sci..

[6] Sunstein C.R., Vermeule A. (2009). Conspiracy Theories: Causes and Cures. J. Political Philos..

[7] Grimes D.R. (2016). On the Viability of Conspiratorial Beliefs. PLoS ONE.

[8] Douglas K.M., Uscinski J.E., Sutton R.M., Cichocka A., Nefes T., Ang C.S., Deravi F. (2019). Understanding Conspiracy Theories. Political Psychol..

[9] van Prooijen J.W., van Vugt M. (2018). Conspiracy Theories: Evolved Functions and Psychological Mechanisms. Perspect. Psychol. Sci..

[10] Prooijen J.V. (2022). Psychological benefits of believing conspiracy theories. Curr. Opin. Psychol..

[11] van Prooijen J.W., Douglas K.M., De Inocencio C. (2018). Connecting the dots: Illusory pattern perception predicts belief in conspiracies and the supernatural. Eur. J. Soc. Psychol..

[12] Biddlestone M., Green R., Cichocka A., Douglas K., Sutton R.M. (2022). A systematic review and meta-analytic synthesis of the motives associated with conspiracy beliefs. PsyArXiv.

[13] Gligorić V., da Silva M.M., Eker S., van Hoek N., Nieuwenhuijzen E., Popova U., Zeighami G. (2021). The usual suspects: How psychological motives and thinking styles predict the endorsement of well-known and COVID-19 conspiracy beliefs. Appl. Cogn. Psychol..

[14] Douglas K.M., Sutton R.M., Cichocka A. (2019). Belief in conspiracy theories: Looking beyond gullibility. The Social Psychology of Gullibility.

[15] Goreis A., Voracek M. (2019). A Systematic Review and Meta-Analysis of Psychological Research on Conspiracy Beliefs: Field Characteristics, Measurement Instruments, and Associations with Personality Traits. Front. Psychol..

[16] Swami V., Coles R., Stieger S., Pietschnig J., Furnham A., Rehim S., Voracek M. (2011). Conspiracist ideation in Britain and Austria: Evidence of a monological belief system and associations between individual psychological differences and real-world and fictitious conspiracy theories. Br. J. Psychol..

[17] Brotherton R., French C.C., Pickering A.D. (2013). Measuring belief in conspiracy theories: The generic conspiracist beliefs scale. Front. Psychol..

[18] Imhoff R., Bertlich T., Frenken M. (2022). Tearing apart the “evil” twins: A general conspiracy mentality is not the same as specific conspiracy beliefs. Curr. Opin. Psychol..

[19] Bruder M., Haffke P., Neave N., Nouripanah N., Imhoff R. (2013). Measuring individual differences in generic beliefs in conspiracy theories across cultures: Conspiracy mentality questionnaire. Front. Psychol..

[20] Leonard M.J., Philippe F.L. (2021). Conspiracy Theories: A Public Health Concern and How to Address It. Front. Psychol..

[21] van Prooijen J.W., Etienne T.W., Kutiyski Y., Krouwel A.P.M. (2023). Conspiracy Beliefs Prospectively Predict Health Behavior and Well-Being during a Pandemic. Psychol. Med..

[22] van Mulukom V., Pummerer L.J., Alper S., Bai H., Čavojová V., Farias J., Kay C.S., Lazarevic L.B., Lobato E.J.C., Marinthe G. (2022). Antecedents and consequences of COVID-19 conspiracy beliefs: A systematic review. Soc. Sci. Med..

[23] Lenhard W., Lenhard A. (2017). Computation of Effect Sizes.

[24] Bogart L.M., Thorburn S. (2005). Are HIV/AIDS conspiracy beliefs a barrier to HIV prevention among African Americans?. J. Acquir. Immune Defic. Syndr..

[25] Earnshaw V.A., Bogart L.M., Klompas M., Katz I.T. (2019). Medical mistrust in the context of Ebola: Implications for intended care-seeking and quarantine policy support in the United States. J. Health Psychol..

[26] Oliver J.E., Wood T. (2014). Medical conspiracy theories and health behaviors in the United States. JAMA Intern. Med..

[27] Pellegrini V., Giacomantonio M., De Cristofaro V., Salvati M., Brasini M., Carlo E., Mancini F., Leone L. (2021). Is Covid-19 a natural event? Covid-19 pandemic and conspiracy beliefs. Pers. Individ. Dif..

[28] Pavela Banai I., Banai B., Mikloušić I. (2022). Beliefs in COVID-19 conspiracy theories, compliance with the preventive measures, and trust in government medical officials. Curr. Psychol..

[29] Giacomantonio M., Pellegrini V., De Cristofaro V., Brasini M., Mancini F. (2022). Expectations about the “Natural Order of Things” and Conspiracy Beliefs about COVID-19. Int. J. Environ. Res. Public Health.

[30] Sallam M., Dababseh D., Eid H., Al-Mahzoum K., Al-Haidar A., Taim D., Yaseen A., Ababneh N.A., Bakri F.G., Mahafzah A. (2021). High Rates of COVID-19 Vaccine Hesitancy and Its Association with Conspiracy Beliefs: A Study in Jordan and Kuwait among Other Arab Countries. Vaccines.

[31] Chayinska M., Uluğ Ö.M., Ayanian A.H., Gratzel J.C., Brik T., Kende A., McGarty C. (2021). Coronavirus conspiracy beliefs and distrust of science predict risky public health behaviours through optimistically biased risk perceptions in Ukraine, Turkey, and Germany. Group Process. Intergroup Relat..

[32] Sallam M., Al-Sanafi M., Sallam M. (2022). A Global Map of COVID-19 Vaccine Acceptance Rates per Country: An Updated Concise Narrative Review. J. Multidiscip. Healthc..

[33] Sallam M., Ghazy R.M., Al-Salahat K., Al-Mahzoum K., AlHadidi N.M., Eid H., Kareem N., Al-Ajlouni E., Batarseh R., Ababneh N.A. (2022). The Role of Psychological Factors and Vaccine Conspiracy Beliefs in Influenza Vaccine Hesitancy and Uptake among Jordanian Healthcare Workers during the COVID-19 Pandemic. Vaccines.

[34] Tomljenovic H., Bubic A., Erceg N. (2020). It just doesn’t feel right—The relevance of emotions and intuition for parental vaccine conspiracy beliefs and vaccination uptake. Psychol. Health.

[35] Baeza-Rivera M.J., Salazar-Fernández C., Araneda-Leal L., Manríquez-Robles D. (2021). Evidencia longitudinal de la intención y conducta vacunatoria contra el COVID-19: Creencias conspirativas y creencias sobre la efectividad de las vacunas como predictores. Psykhe.

[36] Milošević Đorđević J., Mari S., Vdović M., Milošević A. (2021). Links between conspiracy beliefs, vaccine knowledge, and trust: Anti-vaccine behavior of Serbian adults. Soc. Sci. Med..

[37] Romer D., Jamieson K.H. (2020). Conspiracy theories as barriers to controlling the spread of COVID-19 in the U.S. Soc. Sci. Med..

[38] Hughes J.P., Efstratiou A., Komer S.R., Baxter L.A., Vasiljevic M., Leite A.C. (2022). The impact of risk perceptions and belief in conspiracy theories on COVID-19 pandemic-related behaviours. PLoS ONE.

[39] Alsanafi M., Salim N.A., Sallam M. (2023). Willingness to get HPV vaccination among female university students in Kuwait and its relation to vaccine conspiracy beliefs. Hum. Vaccine Immunother..

[40] Bertin P., Nera K., Delouvée S. (2020). Conspiracy Beliefs, Rejection of Vaccination, and Support for hydroxychloroquine: A Conceptual Replication-Extension in the COVID-19 Pandemic Context. Front. Psychol..

[41] Sallam M., Dababseh D., Yaseen A., Al-Haidar A., Taim D., Eid H., Ababneh N.A., Bakri F.G., Mahafzah A. (2020). COVID-19 misinformation: Mere harmless delusions or much more? A knowledge and attitude cross-sectional study among the general public residing in Jordan. PLoS ONE.

[42] Freeman D., Waite F., Rosebrock L., Petit A., Causier C., East A., Jenner L., Teale A.L., Carr L., Mulhall S. (2022). Coronavirus conspiracy beliefs, mistrust, and compliance with government guidelines in England. Psychol. Med..

[43] Tonković M., Dumančić F., Jelić M., Čorkalo Biruški D. (2021). Who Believes in COVID-19 Conspiracy Theories in Croatia? Prevalence and Predictors of Conspiracy Beliefs. Front. Psychol..

[44] Sallam M., Eid H., Awamleh N., Al-Tammemi A.B., Barakat M., Athamneh R.Y., Hallit S., Harapan H., Mahafzah A. (2022). Conspiratorial Attitude of the General Public in Jordan towards Emerging Virus Infections: A Cross-Sectional Study Amid the 2022 Monkeypox Outbreak. Trop. Med. Infect. Dis..

[45] Alsanafi M., Al-Mahzoum K., Sallam M. (2022). Monkeypox Knowledge and Confidence in Diagnosis and Management with Evaluation of Emerging Virus Infection Conspiracies among Health Professionals in Kuwait. Pathogens.

[46] Sallam M., Al-Mahzoum K., Dardas L.A., Al-Tammemi A.B., Al-Majali L., Al-Naimat H., Jardaneh L., AlHadidi F., Al-Salahat K., Al-Ajlouni E. (2022). Knowledge of Human Monkeypox and Its Relation to Conspiracy Beliefs among Students in Jordanian Health Schools: Filling the Knowledge Gap on Emerging Zoonotic Viruses. Medicina.

[47] Jovančević A., Milićević N. (2020). Optimism-pessimism, conspiracy theories and general trust as factors contributing to COVID-19 related behavior—A cross-cultural study. Personal. Individ. Differ..

[48] Salazar-Fernández C., Baeza-Rivera M.J., Villanueva M., Bautista J.A., Navarro R.M., Pino M. (2022). Predictors of COVID-19 Vaccine Intention: Evidence from Chile, Mexico, and Colombia. Vaccines.

[49] Baeza-Rivera M.J., Salazar-Fernández C., Araneda-Leal L., Manríquez-Robles D. (2021). To get vaccinated or not? Social psychological factors associated with vaccination intent for COVID-19. J. Pac. Rim Psychol..

[50] Pertwee E., Simas C., Larson H.J. (2022). An epidemic of uncertainty: Rumors, conspiracy theories and vaccine hesitancy. Nat. Med..

[51] Betsch C., Schmid P., Heinemeier D., Korn L., Holtmann C., Böhm R. (2018). Beyond confidence: Development of a measure assessing the 5C psychological antecedents of vaccination. PLoS ONE.

[52] Larson H.J., Clarke R.M., Jarrett C., Eckersberger E., Levine Z., Schulz W.S., Paterson P. (2018). Measuring trust in vaccination: A systematic review. Hum. Vaccine Immunother..

[53] de Figueiredo A., Simas C., Karafillakis E., Paterson P., Larson H.J. (2020). Mapping global trends in vaccine confidence and investigating barriers to vaccine uptake: A large-scale retrospective temporal modelling study. Lancet.

[54] Solís Arce J.S., Warren S.S., Meriggi N.F., Scacco A., McMurry N., Voors M., Syunyaev G., Malik A.A., Aboutajdine S., Adeojo O. (2021). COVID-19 vaccine acceptance and hesitancy in low- and middle-income countries. Nat. Med..

[55] Betsch C., Schmid P., Verger P., Lewandowsky S., Soveri A., Hertwig R., Fasce A., Holford D., De Raeve P., Gagneur A. (2022). A call for immediate action to increase COVID-19 vaccination uptake to prepare for the third pandemic winter. Nat. Commun..

[56] Mahameed H., Al-Mahzoum K., AlRaie L.A., Aburumman R., Al-Naimat H., Alhiary S., Barakat M., Al-Tammemi A.B., Salim N.A., Sallam M. (2023). Previous Vaccination History and Psychological Factors as Significant Predictors of Willingness to Receive Mpox Vaccination and a Favorable Attitude towards Compulsory Vaccination. Vaccines.

[57] Karlsson L.C., Soveri A., Lewandowsky S., Karlsson L., Karlsson H., Nolvi S., Karukivi M., Lindfelt M., Antfolk J. (2021). Fearing the disease or the vaccine: The case of COVID-19. Pers. Individ. Dif..

[58] Zampetakis L.A., Melas C. (2021). The health belief model predicts vaccination intentions against COVID-19: A survey experiment approach. Appl. Psychol. Health Well Being.

[59] Vogel G., Kupferschmidt K. (2021). New problems erode confidence in AstraZeneca’s vaccine. Science.

[60] Simione L., Vagni M., Gnagnarella C., Bersani G., Pajardi D. (2021). Mistrust and Beliefs in Conspiracy Theories Differently Mediate the Effects of Psychological Factors on Propensity for COVID-19 Vaccine. Front. Psychol..

[61] Sallam M., Dababseh D., Eid H., Hasan H., Taim D., Al-Mahzoum K., Al-Haidar A., Yaseen A., Ababneh N.A., Assaf A. (2021). Low COVID-19 Vaccine Acceptance Is Correlated with Conspiracy Beliefs among University Students in Jordan. Int. J. Environ. Res. Public Health.

[62] Sallam M., Al-Mahzoum K., Eid H., Assaf A.M., Abdaljaleel M., Al-Abbadi M., Mahafzah A. (2021). Attitude towards HPV Vaccination and the Intention to Get Vaccinated among Female University Students in Health Schools in Jordan. Vaccines.

[63] Pilch I., Turska-Kawa A., Wardawy P., Olszanecka-Marmola A., Smołkowska-Jędo W. (2023). Contemporary trends in psychological research on conspiracy beliefs. A systematic review. Front. Psychol..

[64] Ward J.K., Crépin L., Bauquier C., Vergelys C., Bocquier A., Verger P., Peretti-Watel P. (2017). ‘I don’t know if I’m making the right decision’: French mothers and HPV vaccination in a context of controversy. Health Risk Soc..

[65] Salman M., Mallhi T.H., Tanveer N., Shehzadi N., Khan H.M., Ul Mustafa Z., Khan T.M., Hussain K., Mohamed M.S., Maqbool F. (2022). Evaluation of Conspiracy Beliefs, Vaccine Hesitancy, and Willingness to Pay towards COVID-19 Vaccines in Six Countries from Asian and African Regions: A Large Multinational Analysis. Vaccines.

[66] Kennedy J. (2019). Populist politics and vaccine hesitancy in Western Europe: An analysis of national-level data. Eur. J. Public Health.

[67] Bronfin D.R. (2008). Childhood immunization controversies: What are parents asking?. Ochsner J..

[68] Dubé E., Vivion M., MacDonald N.E. (2015). Vaccine hesitancy, vaccine refusal and the anti-vaccine movement: Influence, impact and implications. Expert Rev. Vaccines.

[69] MacDonald N.E. (2015). Vaccine hesitancy: Definition, scope and determinants. Vaccine.

[70] Geiger M., Rees F., Lilleholt L., Santana A.P., Zettler I., Wilhelm O., Betsch C., Böhm R. (2021). Measuring the 7Cs of Vaccination Readiness. Eur. J. Psychol. Assess..

[71] Patwary M.M., Bardhan M., Disha A.S., Hasan M., Haque M.Z., Sultana R., Hossain M.R., Browning M.H.E.M., Alam M.A., Sallam M. (2021). Determinants of COVID-19 Vaccine Acceptance among the Adult Population of Bangladesh Using the Health Belief Model and the Theory of Planned Behavior Model. Vaccines.

[72] Enea V., Eisenbeck N., Carreno D.F., Douglas K.M., Sutton R.M., Agostini M., Bélanger J.J., Gützkow B., Kreienkamp J., Abakoumkin G. (2022). Intentions to be Vaccinated against COVID-19: The Role of Prosociality and Conspiracy Beliefs across 20 Countries. Health Commun..

[73] Bogart L.M., Galvan F.H., Wagner G.J., Klein D.J. (2011). Longitudinal Association of HIV Conspiracy Beliefs with Sexual Risk Among Black Males Living with HIV. AIDS Behav..

[74] Sallam M. (2021). COVID-19 Vaccine Hesitancy Worldwide: A Concise Systematic Review of Vaccine Acceptance Rates. Vaccines.

[75] Salazar-Fernández C., Baeza-Rivera M.J., Manríquez-Robles D. (2022). Escala de creencias hacia las vacunas y hacia la vacuna contra el SARS-CoV-2: Evidencia de sus propiedades psicométricas [Beliefs towards vaccines and SARS-CoV-2 vaccine scales: Evidence of its psychometric properties]. Rev. Médica Chile.

[76] Flora D.B., Curran P.J. (2004). An empirical evaluation of alternative methods of estimation for confirmatory factor analysis with ordinal data. Psychol. Methods.

[77] Marsh H.W., Hau K.-T., Wen Z. (2004). In Search of Golden Rules: Comment on Hypothesis-Testing Approaches to Setting Cutoff Values for Fit Indexes and Dangers in Overgeneralizing Hu and Bentler’s (1999) Findings. Struct. Equ. Model. Multidiscip. J..

[78] Fornell C., Larcker D.F. (1981). Evaluating Structural Equation Models with Unobservable Variables and Measurement Error. J. Mark. Res..

[79] Mackinnon S., Curtis R., O’Connor R. (2022). A tutorial in longitudinal measurement invariance and cross-lagged panel models using lavaan. Meta-Psychology.

[80] Sass D.A., Schmitt T.A., Marsh H.W. (2014). Evaluating model fit with ordered categorical data within a measurement invariance framework: A comparison of estimators. Struct. Equ. Model. Multidiscip. J..

[81] Rutkowski L., Svetina D. (2017). Measurement invariance in international surveys: Categorical indicators and fit measure performance. Appl. Meas. Educ..

[82] Selig J.P., Little T.D., Laursen B.P., Little T.D., Card N.A. (2012). Autoregressive and cross-lagged panel analysis for longitudinal data. Handbook of Developmental Research Methods.

[83] Hamaker E.L., Kuiper R.M., Grasman R. (2015). A critique of the cross-lagged panel model. Psychol. Methods.

[84] Tan S.H., Tan S.B. (2010). The correct interpretation of confidence intervals. Proc. Singap. Healthc..

[85] Sassenberg K., Bertin P., Douglas K.M., Hornsey M.J. (2023). Engaging with conspiracy theories: Causes and consequences. J. Exp. Soc. Psychol..

[86] Sapienza A., Falcone R. (2022). The Role of Trust in COVID-19 Vaccine Acceptance: Considerations from a Systematic Review. Int. J. Environ. Res. Public Health.

[87] Kerekes S., Ji M., Shih S.F., Chang H.Y., Harapan H., Rajamoorthy Y., Singh A., Kanwar S., Wagner A.L. (2021). Differential Effect of Vaccine Effectiveness and Safety on COVID-19 Vaccine Acceptance across Socioeconomic Groups in an International Sample. Vaccines.

[88] Kreps S., Prasad S., Brownstein J.S., Hswen Y., Garibaldi B.T., Zhang B., Kriner D.L. (2020). Factors Associated With US Adults’ Likelihood of Accepting COVID-19 Vaccination. JAMA Netw. Open.

[89] Rosiello D.F., Anwar S., Yufika A., Adam R.Y., Ismaeil M.I., Ismail A.Y., Dahman N.B., Hafsi M., Ferjani M., Sami F.S. (2021). Acceptance of COVID-19 vaccination at different hypothetical efficacy and safety levels in ten countries in Asia, Africa, and South America. Narra J..

[90] Jolley D., Douglas K.M. (2014). The Effects of Anti-Vaccine Conspiracy Theories on Vaccination Intentions. PLoS ONE.

[91] Mattocks K.M., Gibert C., Fiellin D., Fiellin L.E., Jamison A., Brown A., Justice A.C. (2017). Mistrust and Endorsement of Human Immunodeficiency Virus Conspiracy Theories Among Human Immunodeficiency Virus–Infected African American Veterans. Mil. Med..

[92] Moffitt J.D., King C., Carley K.M. (2021). Hunting Conspiracy Theories During the COVID-19 Pandemic. Soc. Media + Soc..

[93] Erokhin D., Yosipof A., Komendantova N. (2022). COVID-19 Conspiracy Theories Discussion on Twitter. Soc. Media + Soc..

[94] ACTIVA Fakenews Desinformación en Chile y LatAm. http://efaidnbmnnnibpcajpcglclefindmkaj/https://aimchile.cl/wp-content/uploads/2023/04/222287_Estudio-Desinformacioin_0329_V1-3-1_compressed-1.pdf.

[95] Borah P., Irom B., Hsu Y.C. (2022). ‘It infuriates me’: Examining young adults’ reactions to and recommendations to fight misinformation about COVID-19. J. Youth Stud..

[96] Chen L., Zhang Y., Young R., Wu X., Zhu G. (2021). Effects of Vaccine-Related Conspiracy Theories on Chinese Young Adults’ Perceptions of the HPV Vaccine: An Experimental Study. Health Commun..

[97] Baron R.A., Branscombe N.R. (2006). Social Psychology.

[98] Kunda Z., Oleson K.C. (1995). Maintaining stereotypes in the face of disconfirmation: Constructing grounds for subtyping deviants. J. Pers. Soc. Psychol..

[99] Bogart L.M., Ojikutu B.O., Tyagi K., Klein D.J., Mutchler M.G., Dong L., Lawrence S.J., Thomas D.R., Kellman S. (2021). COVID-19 Related Medical Mistrust, Health Impacts, and Potential Vaccine Hesitancy among Black Americans Living With HIV. J. Acquir. Immune Defic. Syndr..

[100] Smith A.C., Woerner J., Perera R., Haeny A.M., Cox J.M. (2022). An Investigation of Associations Between Race, Ethnicity, and Past Experiences of Discrimination with Medical Mistrust and COVID-19 Protective Strategies. J. Racial Ethn. Health Disparities.

[101] Cinelli M., Etta G., Avalle M., Quattrociocchi A., Di Marco N., Valensise C., Galeazzi A., Quattrociocchi W. (2022). Conspiracy theories and social media platforms. Curr. Opin. Psychol..

