# Supplementary material for: From Conspiracy to Hesitancy: The Longitudinal Impact of COVID-19 Vaccine Conspiracy Theories on Perceived Vaccine Effectiveness
Source: Vaccines (Basel). 2023 Jun 25;11(7):1150. doi: 10.3390/vaccines11071150 (PMC10386435; doi:10.3390/vaccines11071150)
Supplement: Supplementary file 1 [file vaccines-11-01150-s001.zip › vaccines-2412997-supplementary.pdf]

Supplementary Materials File S1: Pilot Study.

The aim of this pilot study was to estimate the psychometric properties of the conspiracy beliefs about COVID-19 and its vaccine scale. To achieve this aim, we used a non-probabilistic purposive sampling, recruiting 149 university students. Participants of this pilot study were between 19 and 33 years old, lived in Chile during the pandemic, and most identified with the feminine gender (67.78%). More details of participants of the pilot sample are presented in Table S1.

Table S1. Sociodemographic characteristics of the pilot sample.

Variable	Pilot sample<br>Exploratory factor analysis<br>*n* = 149
**Gender identification**	
Feminine	101 (67.78%)
Masculine	44 (29.53%)
Other	4 (2.68%)
Not answered	-
**Age (years)**	
Mean (SD)	21.20 (2.05)

Following providing an e-consent for participation, the participants were asked to answer a sociodemographic questionnaire and followed by the items related to Conspiracy beliefs about COVID-19 and its vaccine. An instrument composed of five items was translated and adapted from Brotherton, *et al.* [1]. These items aimed to assess conspiracy beliefs about the origins of COVID-19 (e.g., bioweapon to destabilize the world or reduce world population size) and COVID-19 vaccine conspiracies (e.g., the vaccines have already been created, the vaccines contain a microchip to be implanted in people, the vaccines were manufactured to control people and to obtain economic profits). These conspiracy items were measured on a 5-point Likert scale, with participants indicating their degree of disagreement (1) or agreement (5) with these items. Higher scores indicated a greater belief in conspiracy theories about COVID-19 and its vaccination.

The pilot study was conducted using Google Forms platform. The participants signed an informed consent to indicate their willingness for participation. The Ethics Committee from the sponsoring university approved this study (N°65/20). Informed consent states the study aims and ensures the anonymity, confidentiality, and contact information of the participants.

Descriptive analyses were performed on the data of the pilot study. Then, we used EFA to explore the structure of the conspiracy beliefs scale (CBS). We employed parallel analysis and principal axis factoring to determine the number of factors to retain and use oblimin rotation. The adequacy of the data matrix was assessed using the Kaiser-Meyer-Olkin (KMO) indicator and Bartlett's sphericity test. KMO should be higher than 0.60, and Bartlett's test should be statistically significant ($p < 0.050$) to assure data adequacy. Due to the ordinal nature of the variables, the McDonald's Omega was used to estimate the scale's internal consistency.

The results for the EFA using the pilot sample ($n = 149$) revealed that the data matrix was suitable to be factorizable, KMO = 0.768, Bartlett's test (10) = 369.162, $p < 0.050$. The 5-items scale showed factor loadings from 0.692 to 0.847 and explained 54.1% of the variance. The one-factor scale showed good reliability ($\omega = 0.870$).

Table S2. Factor loadings of the scale used in the pilot study.

Conspiracy beliefs about COVID-19 and its vaccine scale	M (SD)	Factor loadings ($n = 149$)
1. COVID-19 is a biological weapon created by some countries to destabilize the world	1.92 (1.228)	0.857
2. COVID-19 was created to reduce the world population	1.98 (1.211)	0.799
3. Vaccine against COVID-19 will have a microchip to control persons	1.27 (0.777)	0.535
4. Vaccine against COVID-19 was already created, but they are retaining it to maintain people controlled	1.52 (0.920)	0.743
5. Big Pharma created COVID-19 to benefit from vaccines	1.57 (0.953)	0.702

1. Brotherton, R.; French, C.C.; Pickering, A.D. Measuring belief in conspiracy theories: The generic conspiracist beliefs scale. *Frontiers in psychology* **2013**, *4*, 1-15, doi:10.3389/fpsyg.2013.00279.
